# Transthyretin familial amyloid polyneuropathy impact on health-related quality of life

**DOI:** 10.1186/1750-1172-10-S1-O28

**Published:** 2015-11-02

**Authors:** Mónica Inês, Teresa Coelho, Isabel Conceição, Lara Ferreira, Mamede de Carvalho, João Costa

**Affiliations:** 1 Instituto de Medicina Molecular - Service : Unidade de Fisiologia Clínica e Translacional, 1649-028 Lisboa – Portugal; 2 Centro Hospitalar do Porto - Service : Unidade Corino de Andrade, 4000-436 Porto – Portugal; 3 Centro Hospitalar Lisboa Norte - Service : Serviço de Neurologia, 1649-035 Lisboa – Portugal; 4 Universidade do Algarve - Service : ESGHT, 8005-139 Faro – Portugal

## Background

Transthyretin Familial Amyloid Polyneuropathy (TTR-FAP) is a rare, progressive, debilitating and life-threatening neurodegenerative disease. The purpose of this study was to assess the health-related quality of life (HRQoL) impairment of TTR-FAP disease versus Portuguese general population and to identify individual patient characteristics - such as disease stage – that affects their HRQoL. Literature on TTR-FAP patients HRQoL is scarce at worldwide level and no evidence for Portugal has been published.

## Methods

HRQoL was measured using the validated EuroQoL five dimensions three levels (EQ5D-3L) questionnaire being the index score (utility) calculated trough the Portuguese scoring algorithm. The Portuguese general population reference set (n = 1500) was pooled with TTR-FAP patients specific data extracted from Transthyretin Amyloidosis Outcomes Survey (THAOS) registry. Ordinary Least Squares regression for utility was set to test if being asymptomatic carrier caused HRQoL impairment, conditional in other individual characteristics. Generalized linear models (GLM) were specified for disutility in order to disentangle individual patient characteristics that affect quality of life and quantify the impact. Demographic variables include gender and age. Clinical variables include disease onset (early/late), polyneuropathy disability (PND) score, liver transplant and pharmacologic treatment. Akaike information criteria were used to select the most adequate statistical model.

## Results

In a scale from -0.50 to 1.00 the average utility score was 0.76 (0.25) for general population, 0.823 (0.24) for TTR-FAP asymptomatic carriers (n=525) and 0.50 (0.37) for symptomatic TTR-FAP patients (n = 566). Ordinary Least Squares regression indicated no significant statistical effect on utility for being a TTR-FAP asymptomatic carrier versus general population (p-value 0.54). The GLM was used to detect a significant statistical effect for gender, age and being symptomatic TTR-FAP patient versus general population. Average women aged 44 years and symptomatic TTR-FAP patient, has average 40% impairment on utility versus women aged 44 years from general population. Within TTR-FAP population, individual patient characteristics such as gender, age, disease onset (early/late), polyneuropathy disability (PND) score, liver transplant and pharmacologic treatment were tested for significant statistical effect (p-value <0.005).

## Conclusion

The preference-based utility measures used in this study adequately quantify the large impact of TTR-FAP disease on patient's health-related quality of life and allow discriminating across different TTR-FAP clinical stages, interventions and demographic characteristics. Assuming that these values represent the patients' preferences and the utility associated with their health state, the results presented in this study may be used in future health technologies cost-utility studies.

